# Greater $\dot{V}$O_2peak_ is correlated with greater skeletal muscle deoxygenation amplitude and hemoglobin concentration within individual muscles during ramp‐incremental cycle exercise

**DOI:** 10.14814/phy2.13065

**Published:** 2016-12-16

**Authors:** Dai Okushima, David C. Poole, Thomas J. Barstow, Harry B. Rossiter, Narihiko Kondo, T. Scott Bowen, Tatsuro Amano, Shunsaku Koga

**Affiliations:** ^1^ Applied Physiology LaboratoryKobe Design UniversityKobeJapan; ^2^ Departments of Kinesiology and Anatomy and PhysiologyKansas State UniversityManhattanKansas; ^3^ Rehabilitation Clinical Trials CenterDivision of Respiratory & Critical Care Physiology & MedicineLos Angeles Biomedical Research Institute at Harbor‐UCLA Medical CenterTorranceCalifornia; ^4^ Laboratory for Applied Human PhysiologyGraduate School of Human Development and EnvironmentKobe UniversityKobeJapan; ^5^ Department of Internal Medicine & CardiologyHeart CenterLeipzig UniversityLeipzigGermany; ^6^ Faculty of EducationNiigata UniversityNiigataJapan

**Keywords:** Diffusive oxygen potential, fractional oxygen extraction, peak oxygen uptake, time‐resolved NIRS

## Abstract

It is axiomatic that greater aerobic fitness ($\dot{V}$O_2peak_) derives from enhanced perfusive and diffusive O_2_ conductances across active muscles. However, it remains unknown how these conductances might be reflected by regional differences in fractional O_2_ extraction (i.e., deoxy [Hb+Mb] and tissue O_2_ saturation [S_t_O_2_]) and diffusive O_2_ potential (i.e., total[Hb+Mb]) among muscles spatially heterogeneous in blood flow, fiber type, and recruitment (*vastus lateralis*, VL; *rectus femoris*, RF). Using quantitative time‐resolved near‐infrared spectroscopy during ramp cycling in 24 young participants ($\dot{V}$O_2peak_ range: ~37.4–66.4 mL kg^−1^ min^−1^), we tested the hypotheses that (1) deoxy[Hb+Mb] and total[Hb+Mb] at $\dot{V}$O_2peak_ would be positively correlated with $\dot{V}$O_2peak_ in both VL and RF muscles; (2) the pattern of deoxygenation (the deoxy[Hb+Mb] slopes) during submaximal exercise would not differ among subjects differing in $\dot{V}$O_2peak_. Peak deoxy [Hb+Mb] and S_t_O_2_ correlated with $\dot{V}$O_2peak_ for both VL (*r *=* *0.44 and −0.51) and RF (*r *=* *0.49 and −0.49), whereas for total[Hb+Mb] this was true only for RF (*r *=* *0.45). Baseline deoxy[Hb+Mb] and S_t_O_2_ correlated with $\dot{V}$O_2peak_ only for RF (*r *=* *−0.50 and 0.54). In addition, the deoxy[Hb+Mb] slopes were not affected by aerobic fitness. In conclusion, while the pattern of deoxygenation (the deoxy[Hb+Mb] slopes) did not differ between fitness groups the capacity to deoxygenate [Hb+Mb] (index of maximal fractional O_2_ extraction) correlated significantly with $\dot{V}$O_2peak_ in both RF and VL muscles. However, only in the RF did total[Hb+Mb] (index of diffusive O_2_ potential) relate to fitness.

## Introduction

For large muscle mass exercise such as cycling or running the maximum pulmonary O_2_ flux ($\dot{V}$O_2max_) is the product of whole‐body perfusive ($\dot{Q}$O_2_ = cardiac output [$\dot{Q}$] × arterial O_2_ concentration [CaO_2_]) and diffusive (muscle transcapillary) O_2_ conductances. The Fick relationship ($\dot{V}$O_2_ = $\dot{Q}$× [CaO_2_‐C$\overset{¯}{v}$O_2_] where $\overset{¯}{v}$ is mixed venous), as measured across the lungs, provides a volume‐weighted average of fractional O_2_ extractions across all body compartments (Benson et al. 2013; Fick 1870); each with its own unique $\dot{Q}$O_2_/$\dot{V}$O_2_ ratio, and thus effluent venous blood O_2_ concentration. Thus, without discrete measurements within each tissue, assessments across the lungs provide little insight into $\dot{Q}$O_2_‐to‐$\dot{V}$O_2_ matching and the roles of perfusional and diffusional O_2_ transport within each tissue.

During progressive exercise from rest to $\dot{V}$O_2max_ whole‐body O_2_ extraction increases approximately hyperbolically from 25% to 80% or more according to the approximation (Stringer et al. 2005): (CaO_2_‐CvO_2_) = (20 × $\dot{V}$O_2_)/(1 + $\dot{V}$O_2_) (Whipp and Ward 1982). This relationship supports the observations that: individuals with greater $\dot{V}$O_2max_ demonstrate a greater fractional O_2_ extraction than their less aerobically‐fit counterparts in both health and pathophysiological conditions (Grassi et al. 2007; Porcelli et al. 2010; Salvadego et al. 2016) and endurance training increases both fractional O_2_ extraction and $\dot{V}$O_2max_, although not necessarily in linear proportion (Klausen et al. 1982; Roca et al. 1992; Beere et al. 1999). To date, in human muscles, our understanding of these relationships is based overwhelmingly upon measurements made across the whole exercising limb. Within small regions of active muscle, data from both positron emission tomography imaging (Koga et al. 2014; Heinonen et al. 2015) and near‐infrared spectroscopy (NIRS) investigations (Koga et al. 2007, 2011, 2014; Okushima et al. 2015) indicated a substantial heterogeneity of the $\dot{Q}$O_2_/$\dot{V}$O_2_ ratio. This heterogeneity likely arises, in part, from differential motor unit recruitment (Chin et al. 2011) and fiber‐type expression (Johnson et al. 1973). In addition, the degree to which $\dot{Q}$O_2_ and $\dot{V}$O_2_ are well matched within muscles, appears to be greater in trained compared with untrained men (Kalliokoski et al. 2005). However, during cycling exercise from rest to $\dot{V}$O_2peak_, whether or not regional $\dot{Q}$O_2_/$\dot{V}$O_2_ ratio differs among individuals differing in $\dot{V}$O_2peak_ remains unclear. In whole body and thigh muscle $\dot{Q}$O_2_ and $\dot{V}$O_2_ increase linearly with work rate (Knight et al. 1993; Gifford et al. 2016) and therefore the muscle deoxygenation/fractional O_2_ extraction will reach a greater peak in fitter individuals (Ferreira et al. 2006; Gifford et al. 2016; Roca et al. 1992). Based upon this logic, we hypothesize that the different regions of active muscle assessed using NIRS will have a similar overall profile of deoxygenation but the regional peak $\dot{Q}$O_2_/$\dot{V}$O_2_ values (i.e., deoxy[Hb+Mb]) will correlate with whole‐body $\dot{V}$O_2peak_.

Recently we employed a time‐resolved (TRS) NIRS system, with adipose tissue thickness correction, to determine muscle optical coefficients and therefore measure absolute deoxy‐ and total‐[Hb+Mb] and tissue O_2_ saturation (S_t_O_2_) (Koga et al. 2011, 2015a; Okushima et al. 2015), which are not impacted significantly by skin blood flow changes (Koga et al. 2015b). Although TRS‐NIRS variables do not directly measure muscle $\dot{V}$O_2_ and $\dot{Q}$O_2_, evidence from direct measurements during muscle contractions suggest that deoxy[Hb+Mb] is an index of regional fractional O_2_ extraction in the face of presiding blood flow (i.e., microvascular O_2_ partial pressure (Koga et al. 2012)) and femoral venous O_2_ saturation (Vogiatzis et al. 2015; Sun et al. 2016). Moreover, the total[Hb+Mb] is an index of microvascular blood volume (Ijichi et al. 2005), which reflects local O_2_ diffusing capacity (Groebe and Thews 1990). The purpose of this study was to use the TRS‐NIRS technology to investigate O_2_ perfusive ($\dot{Q}$O_2_/$\dot{V}$O_2_) and diffusive profiles across the range of achievable power output in individuals ranging broadly in aerobic capacity. We addressed the following specific hypothesis during maximal ramp cycle ergometry: (1) deoxy[Hb+Mb] and total[Hb+Mb] in both VL and RF muscles at peak power output would be positively correlated with $\dot{V}$O_2peak_, and (2) the pattern of deoxygenation (the deoxy[Hb+Mb] slopes) during submaximal exercise would not differ among subjects differing in $\dot{V}$O_2peak_.

## Methods

### Subjects

Twenty‐four young males participated in this study (age, 22 ± 2 years; height, 174 ± 5 cm; weight, 62 ± 5 kg). All participants were nonsmokers and free of known cardiovascular, respiratory, and metabolic disease. This study was approved by the Human Subjects Committee of Kobe Design University, in compliance with the Declaration of Helsinki. Each participant signed an informed consent form after explanation of experimental procedures and the potential risks and benefits of taking part.

### Experimental procedure

Participants started the experimental procedure at least 2 h postprandial and refrained from caffeine, alcohol, and intense exercise for 24 h before testing. All experiments were conducted in environmental chambers (SR‐3000, Nagano science, Osaka, Japan) maintained at the ambient temperature of 21–24°C and relative humidity of 40–60%, in order to maintain ambient conditions for the studies across Japanese seasons.

Exercise testing was completed on an electromagnetically braked cycling ergometer (XL‐75III, Combi, Tokyo, Japan) in the upright position. The exercise protocol began with 2 min of rest, followed by 4 min of 20 W baseline exercise and then a ramp‐incremental exercise test. The ramp increase was 20 W min^−1^. Participants were asked to maintain pedaling frequency at 60 rpm throughout the exercise test, and the test was terminated when participants could no longer maintain 60 rpm despite strong verbal encouragement. At the limit of tolerance the power output was reduced to 20 W and the participants were monitored for at least 6 min of active recovery.

### Measurement

#### Pulmonary oxygen uptake ($\dot{V}$O_2_)

Gas exchange measurement methods were the same as in previous studies (Koga et al. 2011; Bowen et al. 2013; Spencer et al. 2014; Okushima et al. 2015). The breath‐by‐breath gas exchange system (AE‐300S, Minato‐Medical, Osaka, Japan) was calibrated according to the manufacturer's recommendation before each exercise test. Participants breathed through a low resistance mouthpiece containing a hot‐wire flowmeter for measurement of inspiratory and expiratory flows and volumes. Inspired and expired gases were continuously sampled from the mouth and O_2_ and CO_2_ fractional concentration was measured by fast‐responding paramagnetic and infrared analyzers, respectively. Gas volume and concentration signals were time‐aligned to account for the time lag between the signals to calculate $\dot{V}$O_2_ and $\dot{V}$CO_2_ on a breath‐by‐breath basis.

#### Near‐infrared spectroscopy

Absolute values of oxygenated (oxy[Hb+Mb]), deoxygenated (deoxy[Hb+Mb]), and total hemoglobin and myoglobin concentration (total[Hb+Mb]) were sampled from the mid‐belly of the *vastus lateralis* (VL) and *rectus femoris* (RF) on the dominant leg by two TRS‐NIRS instruments (TRS‐20, Hamamatsu photonics KK, Hamamatsu, Japan). These muscles were selected because they represent major contributors to power generation in cycle ergometry, have a somewhat different fiber‐type composition (i.e., superficial‐deep Type I %, VL 38–47 %, RF 30–42 %, Johnson et al. 1973) and are differentially recruited during maximal ramp exercise (Chin et al. 2011).

Before applying the probes, the skin under the probes was shaved. The NIRS optodes were housed in black rubber holders and fixed to the skin with adhesive tape to minimize incidental movement and intrusion of ambient light on the NIRS detectors. The interoptode spacing between irradiation and detection probes was 3 cm for all measurement sites. The sample rate was set to 1 Hz.

#### Ultrasonographic imaging

Adipose tissue thickness (ATT) at each NIRS site was measured using B‐mode Doppler ultrasound (Logiq 400, GE‐Yokogawa Medical Systems, Tokyo, Japan). Ultrasound images were collected with care to prevent pressure and distortion of the thickness of the skin and adipose tissues under the probe.

### Data analysis

Peak pulmonary $\dot{V}$O_2_ ($\dot{V}$O_2peak_) was determined as the mean value of the last 30 sec during the ramp exercise test. Participants were grouped into those with low aerobic capacity (LO: *n* = 8, ≤45 mL kg^−1^ min^−1^), mid‐range (MID, *n* = 9, 46–55 mL kg^−1^ min^−1^) and those with high aerobic capacity (HI, *n* = 7, ≥56 mL kg^−1^ min^−1^) using similar reference ranges as in a previous study (Gifford et al. 2016). Analysis of deoxy‐ and total[Hb+Mb] was performed after correction for ATT to a thickness of 0 mm using linear regression of the relationship between total[Hb+Mb] and ATT at rest (Bowen et al. 2013; Okushima et al. 2015) (total [Hb+Mb] = 15.7 × [ATT] + 201, *r* ^*2*^ = 0.801, *P *<* *0.001). For NIRS measurements (deoxy‐ and total[Hb+Mb] and S_t_O_2_), 1 Hz optical measurements were averaged over 6 data points, resulting in one NIRS datum calculation every 6 sec. The baseline of each NIRS measurement was calculated as the mean value of 60 sec prior to the start of ramp exercise. The absolute value of each NIRS measurement during ramp exercise was calculated at 20 W, 50 W and each 50 W increment thereafter, as well as for each 10 % of peak power (W_peak_) increment between 10 and 100 %W_peak_. NIRS variable amplitudes were derived from the difference between baseline and the mean value of the final 18 sec of ramp exercise. The slope of NIRS variables across absolute power (i.e., watt) was determined from linear regression in two regions of the response: below 50 %W_peak_ and above 70 %W_peak_ (Okushima et al. 2015).

### Statistical analysis

All values were expressed as mean ± standard deviation. Pearson product moment correlation was calculated to quantify the relationship between NIRS variables and $\dot{V}$O_2peak_. Comparisons of $\dot{V}$O_2peak_ and W_peak_ were analyzed by one‐way ANOVA with the main effect of fitness (LO, MID, HI). Comparisons of baseline and amplitude in each NIRS variable and ATT were analyzed by two‐way repeated‐measures ANOVA, with main effects of aerobic fitness and muscle site (VL, RF). Comparison of the temporal profile for NIRS variables was analyzed by two‐way repeated‐measures ANOVA, with main effects of aerobic fitness and exercise intensity (normalized power output: every 10 %W_peak_ from 10 to 100 %W_peak_; absolute power output: 20 W, peak power and every 50 W from 50 to 200 W). Comparison of the slopes of each NIRS variable was made by two‐way repeated‐measures ANOVA, with main effects of aerobic fitness and exercise intensity (<50 %W_peak_, >70 %W_peak_). A significant F ratio was analyzed using Bonferroni's post‐hoc test. Effect size (ES: using Cohen's *d*) and statistical power (1‐*β*) were also calculated for the comparison of each NIRS variable. Significance was accepted at *P *<* *0.05.

## Results

By design, participants achieved a wide range of peak power (W_peak_), ranging from 204 to 385 W, and a $\dot{V}$O_2peak_, ranging from 37.4 to 66.4 mL kg^−1^ min^−1^ (Table 1). ATT averaged 4.0 ± 1.2 mm and 5.5 ± 1.8 mm for the VL and RF, respectively, and was significantly less for the VL than the RF muscle (Table 1). However, ATT was not significantly different across participants differing in $\dot{V}$O_2peak_.

**Table 1:** Participant's characteristics

	W_peak_ (W)	$\dot{V}$O_2peak_ (mL kg^−1^ min^−1^)	Adipose tissue thickness (mm)	
			VL	RF
LO (*n* = 8)	240 ± 30	40.1 ± 2.7	4.8 ± 1.6	6.7 ± 2.0^c^
MID (*n* = 9)	285 ± 30^a^	50.3 ± 2.8^a^	3.6 ± 1.0	4.8 ± 1.5^c^
HI (*n* = 7)	325 ± 34^a^^,^ b	61.0 ± 3.6^a^^,^ b	3.7 ± 0.5	5.0 ± 1.4^c^
All (*n* = 24)	282 ± 45	50.0 ± 8.9	4.0 ± 1.2	5.5 ± 1.8

Data are grouped by aerobic capacity ($\dot{V}$O_2peak_): low (LO, ≤45 mL kg^−1^ min^−1^); middle (MID, 46‐55 mL kg^−1^ min^−1^); and high (HI, ≥56 mL kg^−1^ min^−1^).

a Indicates a significant difference from LO (*P *<* *0.05).

b Indicates a significant difference from MID (*P *<* *0.05).

c Indicates a significant difference between VL and RF (*P *<* *0.05).

**Figure 1:**
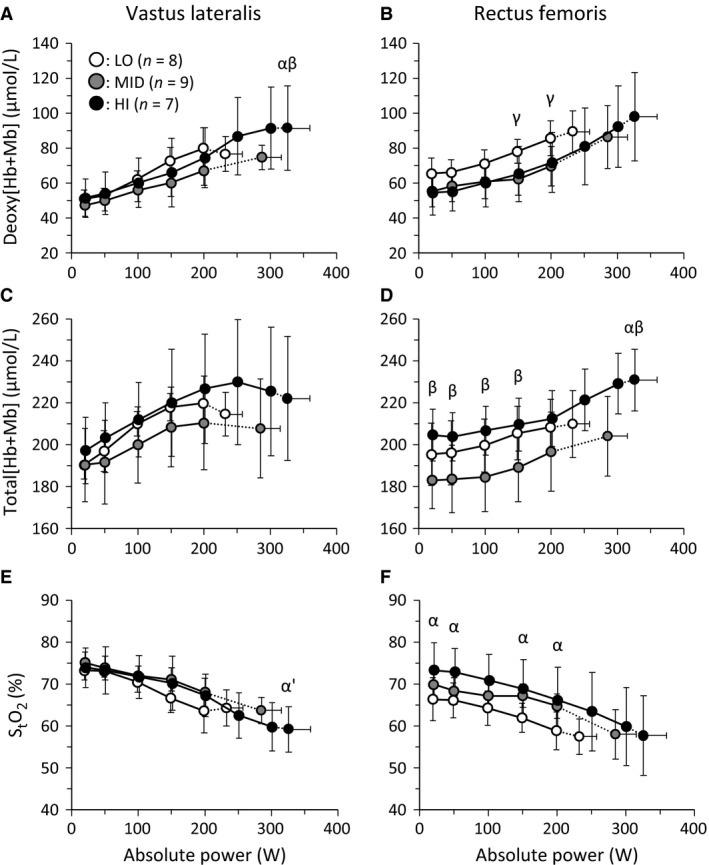
The mean responses of NIRS variables for low (LO, $\dot{V}$O_2peak_ ≤45 mL kg^−1^ min^−1^, open), middle (MID, 46–55 mL kg^−1^ min^−1^, gray), and high aerobic capacity (HI, ≥56 mL kg^−1^ min^−1^, filled) across absolute power. Left and right panels show the NIRS variables in VL and RF muscle, respectively. “*α*”, “*β*”, and “*γ*” indicate the significant difference (*P *<* *0.05) between HI and LO, HI and MID, and LO and MID, respectively. “*α′*” indicates the tendency of significant difference (*P *=* *0.05) between HI and LO.

**Figure 2:**
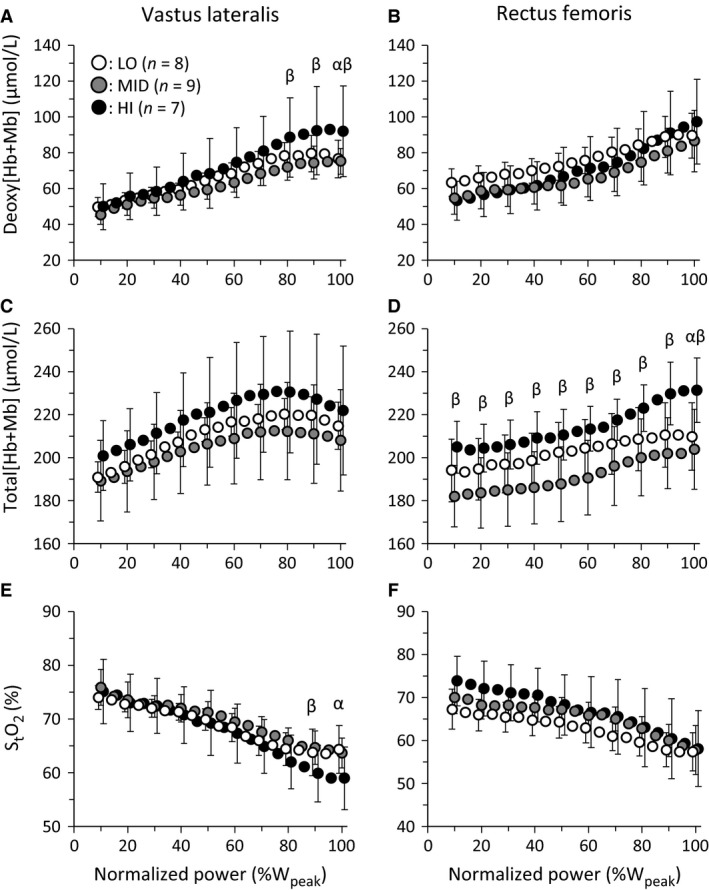
The mean responses of NIRS variables for low (LO, $\dot{V}$O_2peak_ ≤45 mL kg^−1^ min^−1^, open), middle (MID, 46–55 mL kg^−1^ min^−1^, gray), and high aerobic capacity (HI, ≥56 mL kg^−1^ min^−1^, filled) across normalized power. Left and right panels show the NIRS variables in VL and RF muscle, respectively. “*α*” and “β” indicate the significant differences (*P *<* *0.05) between HI and LO, and HI and MID, respectively.

Across absolute submaximal power outputs up to 200 W, deoxy[Hb+Mb] was greater in LO than MID at 150 and 200 W (*P *<* *0.05) for RF muscle, whereas total[Hb+Mb] and S_t_O_2_ were greater in HI than MID (*P *<* *0.05, except 200 W) and LO (*P *<* *0.05, except 100 W) for only the RF muscle (Fig. 1). At peak power output, deoxy[Hb+Mb] in VL and total[Hb+Mb] in RF were greater in HI than LO and MID (*P *<* *0.05), while S_t_O_2_ in VL tended to be less in HI compared to LO (*P *=* *0.05). When exercise intensity was normalized (Fig. 2), deoxy[Hb+Mb] was greater in HI than LO at 100 %W_peak_ and MID at 80–100 %W_peak_ in the VL (*P *<* *0.05, Fig. 2). In addition, S_t_O_2_ was less in HI than LO at 100 %W_peak_ and MID at 90 %W_peak_ in the VL (*P *<* *0.05). In the RF muscle, total[Hb+Mb] was greater in HI than LO at 100 %W_peak_ and greater than MID at all intensities (*P *<* *0.05).

**Figure 3:**
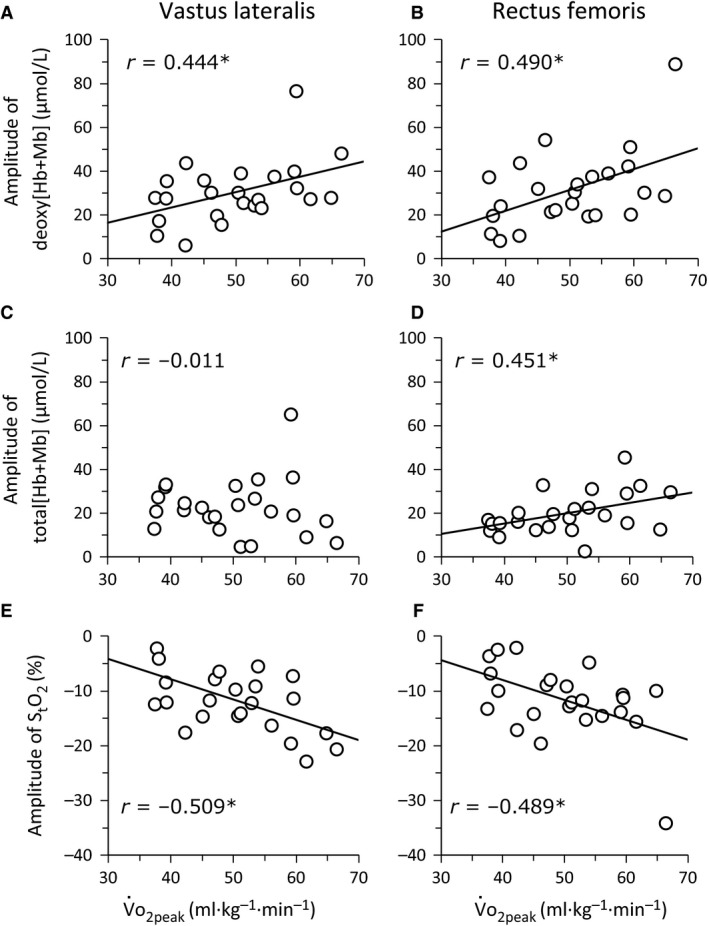
Relationship of the peak amplitude of muscle deoxygenation (deoxy[Hb+Mb]), total tissue hemoglobin and myoglobin concentration (total[Hb+Mb]), and tissue O_2_ saturation (S_t_O_2_) with $\dot{V}$O_2peak_ in the *vastus lateralis* (left panel) and *rectus femoris* muscles (right panel) during ramp‐incremental cycle ergometry. *P <* *0.05.

**Figure 4:**
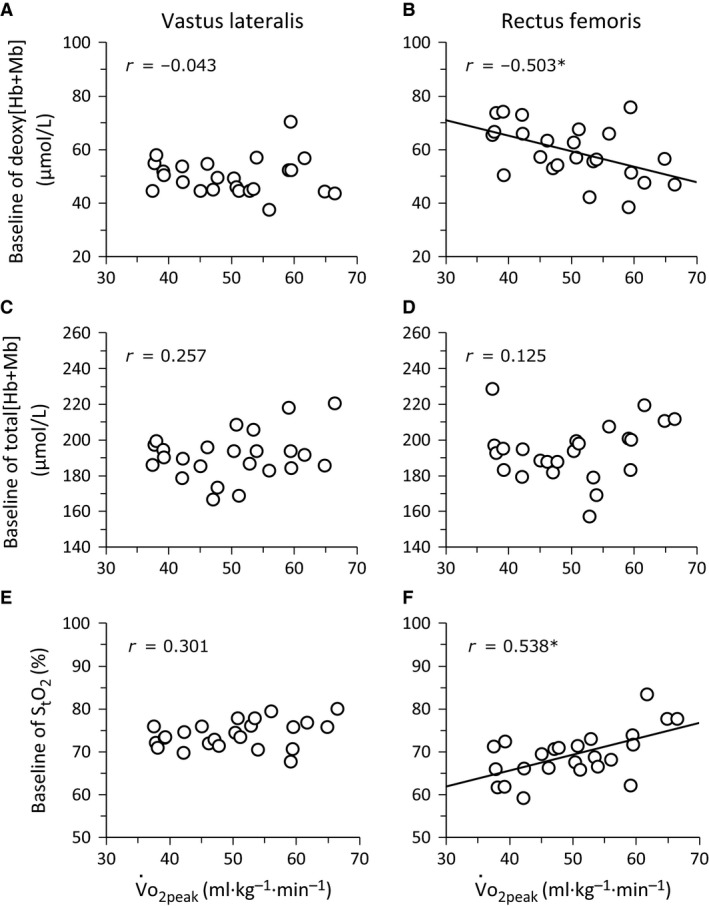
Relationship of the baseline in muscle deoxygenation (deoxy[Hb+Mb]), total tissue hemoglobin and myoglobin concentration (total[Hb+Mb]), and tissue O_2_ saturation (S_t_O_2_) with $\dot{V}$O_2peak_ in the *vastus lateralis* (left panel) and *rectus femoris* muscles (right panel) during ramp‐incremental cycle ergometry. *P <* *0.05.

The VL muscle evinced a lower baseline deoxy[Hb+Mb] (*P *<* *0.001, ES = 0.89, 1‐*β* = 0.99, Table 2) and greater baseline S_t_O_2_ (*P *<* *0.001, ES = 1.05, 1‐*β* = 0.97) compared to the RF muscle, in all $\dot{V}$O_2peak_ groups. Baseline total[Hb+Mb] was greater in HI than MID for both RF and VL muscles (*P *=* *0.019, ES = 1.04, 1‐*β* = 0.81). The exercise‐induced increase in deoxy[Hb+Mb] (*P *=* *0.041, ES = 1.08, 1‐*β* = 0.81) and decrease in S_t_O_2_ (*P *=* *0.035, ES = 1.14, 1‐*β* = 0.92) were greater in HI than LO in both VL and RF muscles. The deoxy[Hb+Mb] and S_t_O_2_ amplitudes were significantly correlated with $\dot{V}$O_2peak_ (Fig. 3) in both VL and RF muscles. At each muscle site, the deoxy[Hb+Mb] and S_t_O_2_ baselines (negative correlation) and the amplitude of total[Hb+Mb] (positive correlation) were significantly correlated with $\dot{V}$O_2peak_ in RF (Figs. 3 and 4) but not VL.

**Table 2:** Comparison of baseline and peak amplitude in deoxy‐ and total[Hb+Mb] and tissue O_2_ saturation (S_t_O_2_) in the *vastus lateralis* (VL) and *rectus femoris* (RF) muscle during incremental cycle ergometry

	VL	RF
Baseline		
Deoxy[Hb+Mb] (*μ*mol/L)		
LO (*n* = 8)	50.8 ± 4.8	66.0 ± 8.4^c^
MID (*n* = 9)	48.6 ± 4.6	57.0 ± 7.3^c^
HI (*n* = 7)	51.2 ± 10.7	54.8 ± 12.6^c^
Total[Hb+Mb] (*μ*mol/L)		
LO (*n* = 8)	190.3 ± 6.8	195.1 ± 15.0
MID (*n* = 9)	188.3 ± 15.4	183.9 ± 13.9
HI (*n* = 7)	196.9 ± 15.9^b^	204.9 ± 11.6^b^
S_t_O_2_ (%)		
LO (*n* = 8)	73.3 ± 2.2	66.1 ± 4.8^c^
MID (*n* = 9)	74.1 ± 2.7	69.1 ± 2.6^c^
HI (*n* = 7)	75.2 ± 4.5	73.6 ± 7.0^c^
Peak amplitude		
Deoxy[Hb+Mb] (*μ*mol/L)		
LO (*n* = 8)	25.6 ± 13.2	23.4 ± 13.2
MID (*n* = 9)	26.1 ± 6.8	29.5 ± 11.3
HI (*n* = 7)	41.4 ± 17.1^a^	43.0 ± 22.7^a^
Total [Hb+Mb] (*μ*mol/L)		
LO (*n* = 8)	24.4 ± 6.5	14.8 ± 3.5
MID (*n* = 9)	19.8 ± 11.0	19.5 ± 9.3
HI (*n* = 7)	24.9 ± 20.3	26.4 ± 11.3
S_t_O_2_ (%)		
LO (*n* = 8)	−8.9 ± 6.3	−8.6 ± 5.8
MID (*n* = 9)	−10.1 ± 3.2	−11.3 ± 4.3
HI (*n* = 7)	−16.5 ± 5.5^a^	−15.7 ± 8.4^a^

Data are grouped by aerobic capacity ($\dot{V}$O_2peak_): low (LO, ≤45 mL kg^−1^ min^−1^); middle (MID, 46–55 mL kg^−1^ min^−1^); and high (HI, ≥56 mL kg^−1^ min^−1^).

a Indicates a significant difference from LO (*P *<* *0.05).

b Indicates a significant difference from MID (*P *<* *0.05).

c Indicates a significant difference between VL and RF (*P *<* *0.01).

In each aerobic fitness group, deoxy[Hb+Mb] increased systematically during exercise up to $\dot{V}$O_2peak_ in the RF muscle, but in the VL muscle deoxy[Hb+Mb] plateaued above 70 %W_peak_ (Figs. 1 and 2, Table 3). Thus, in the VL muscle, deoxy[Hb+Mb] increased far more slowly above 70 %W_peak_ than below 50 %W_peak_ irrespective of $\dot{V}$O_2peak_ (*P *=* *0.033, ES = 0.54, 1‐*β* = 0.72). In contrast, in the RF muscle deoxy[Hb+Mb] increased more rapidly above 70 %W_peak_ than below 50 %W_peak_ (*P *<* *0.001, ES = 1.26, 1‐*β* = 0.99). The total[Hb+Mb] increased systematically to $\dot{V}$O_2peak_ in the RF muscle but decreased above 70 %W_peak_ in the VL muscle independent of aerobic capacity group (*P *<* *0.001, ES = 1.55, 1‐*β* = 1.00). The S_t_O_2_ in the VL decreased systematically up to $\dot{V}$O_2peak_, whereas the S_t_O_2_ in RF muscle decreased more rapidly above 70 %W_peak_ than below 50 %W_peak_ irrespective of aerobic capacity group (*P *<* *0.001, ES = 1.21, 1‐*β* = 0.99).

**Table 3:** Comparison of the slope per watt of deoxy‐ and total[Hb+Mb] and S_t_O_2_ in the *vastus lateralis* (VL) and *rectus femoris* (RF) muscle during incremental cycle ergometry

	VL		RF	
	<50 %W_peak_	>70 %W_peak_	<50 %W_peak_	>70 %W_peak_
Deoxy[Hb+Mb] (*μ*mol/L/W)				
LO (*n* = 8)	0.15 ± 0.06	0.05 ± 0.13^a^	0.09 ± 0.03	0.16 ± 0.10^a^
MID (*n* = 9)	0.11 ± 0.05	0.09 ± 0.05^a^	0.06 ± 0.03	0.21 ± 0.11^a^
HI (*n* = 7)	0.14 ± 0.08	0.13 ± 0.12^a^	0.09 ± 0.05	0.25 ± 0.10^a^
Total[Hb+Mb] (*μ*mol/L/W)				
LO (*n n* = 8)	0.24 ± 0.06	−0.04 ± 0.11^a^	0.10 ± 0.06	0.06 ± 0.07
MID (*n* = 9)	0.15 ± 0.05	−0.04 ± 0.09^a^	0.04 ± 0.05	0.08 ± 0.10
HI (*n* = 7)	0.17 ± 0.11	−0.09 ± 0.16^a^	0.05 ± 0.06	0.17 ± 0.12
S_t_O_2_ (%/W)				
LO (*n* = 8)	−0.04 ± 0.03	−0.03 ± 0.06	−0.03 ± 0.01	−0.06 ± 0.04^a^
MID (*n* = 9)	−0.03 ± 0.02	−0.05 ± 0.02	−0.02 ± 0.02	−0.09 ± 0.05^a^
HI (*n* = 7)	−0.04 ± 0.02	−0.07 ± 0.03	−0.04 ± 0.02	−0.08 ± 0.02^a^

Data are grouped by aerobic capacity ($\dot{V}$O_2peak_): low (LO, ≤45 mL kg^−1^ min^−1^); middle (MID, 46–55 mL kg^−1^ min^−1^); and high (HI, ≥56 mL kg^−1^ min^−1^).

a Indicates a significant difference from <50 %W_peak_ (*P *<* *0.05).

## Discussion

This investigation resolved the magnitude and pattern of $\dot{Q}$O_2_/$\dot{V}$O_2_ matching and O_2_ diffusive potential in the VL and RF muscles across groups of individuals ranging broadly in aerobic capacity using quantitative TRS‐NIRS during ramp‐incremental cycling exercise. The principal original findings are: (1) the magnitude of absolute deoxy[Hb+Mb] increase (i.e., the amplitude of the exercise‐induced reduction in $\dot{Q}$O_2_/$\dot{V}$O_2_) and S_t_O_2_ decrease were correlated with $\dot{V}$O_2peak_ in both VL and RF muscles; (2) a high baseline deoxygenation and S_t_O_2_ in the RF muscle (i.e., low preexercise $\dot{Q}$O_2_/$\dot{V}$O_2_) was negatively associated with $\dot{V}$O_2peak_; (3) the magnitude of increase in the total[Hb+Mb] (i.e., the exercise‐induced increase in O_2_ diffusive potential) was positively correlated with $\dot{V}$O_2peak_ in the RF muscle only; (4) the rate of change in each NIRS variable across absolute work rates was not related to aerobic fitness (i.e., $\dot{V}$O_2peak_).

### The absolute peak amplitude and baseline of muscle deoxygenation

The peak absolute amplitude of deoxy[Hb+Mb] increase (positively) and S_t_O_2_ decrease (negatively) were correlated with $\dot{V}$O_2peak_ for both muscle sites (Table 2, Figs. 1, 2, 3) and this represents the first demonstration of this relationship with technology capable of measuring absolute deoxy[Hb+Mb] together with ATT correction. This is consistent with the notion that the capacity for O_2_ extraction between the capillary and myocyte relates to the aerobic capacity of active muscle. Previous studies have reported that the systemic arteriovenous O_2_ difference and O_2_ extraction capacity (Murias et al. 2010) and leg O_2_ extraction capacity (Roca et al. 1992; Sala et al. 1999; Proctor et al. 2001) are associated with $\dot{V}$O_2max_. These data extend that notion to the level of individual muscles. Blomstrand et al. (1997) and Robinson et al. (1994) reported that a greater $\dot{V}$O_2max_ in human and rat muscle is attributed to a greater O_2_ flux capacity, consequent to a greater muscle mitochondrial enzyme activity. The present results suggest that the increase in the peak amplitude of muscle deoxygenation reflects the peripheral adaptation of fractional O_2_ extraction to exercise at each muscle site. In addition, this was not different between VL and RF muscles, irrespective of $\dot{V}$O_2peak_ (Table 2). Thus, the amplitude of muscle deoxygenation at $\dot{V}$O_2peak_ may be limited to a similar magnitude among active muscle sites.

Baseline deoxy[Hb+Mb] were greater in RF compared with VL independent of $\dot{V}$O_2peak_ (Table 2), and was associated with $\dot{V}$O_2peak_ in RF but not in VL (Fig. 4), extending our previous findings (Chin et al. 2011; Okushima et al. 2015). This suggests that less aerobically‐fit individuals may have a lower $\dot{Q}$O_2_/$\dot{V}$O_2_ of leg muscles at rest or during very low exercise intensities. A low $\dot{Q}$O_2_/$\dot{V}$O_2_ at baseline is consistent with a small intercept for the $\dot{Q}$O_2_/$\dot{V}$O_*2*_ relationship, which predisposes capillary‐myocyte O_2_ flux to be highly sensitive to the adjustment in convective O_2_ delivery: only a small “underperfusion” would rapidly limit capillary PO_2_ (Ferreira et al. 2006; Benson et al. 2013). However, the mechanistic bases for this effect, be they structural (e.g., muscle fiber composition and vascular bed) and/or functional (e.g., regulation of O_2_ distribution and muscle recruitment), remain unclear at present.

### The absolute peak amplitude and baseline of total[Hb+Mb]

The peak absolute amplitude of total[Hb+Mb] for RF, but not for VL, was significantly related to $\dot{V}$O_*2*peak_ (Fig. 3). Our previous study reported that RF activation increased slower than that of the VL and was subsequently speeded further from high submaximal exercise intensities up to $\dot{V}$O_*2*peak_ (Chin et al. 2011). Another study reported that in the VL muscle there was a relationship between the total[Hb+Mb] plateau onset and the steep reduction in the EMG mean power frequency (MPF) (Boone et al. 2016), where the MPF reduction is thought to reflect a reduction in fast twitch fiber activity related to muscle fatigue (Komi and Tesch 1979) (although this interpretation of MPF is controversial). These findings support that the RF muscle increases capillary O_2_ diffusional capacity by longitudinal capillary recruitment (Poole et al. 2013), whereas the VL muscle is limited in this regard. Thus in the RF muscle the relationship of the absolute total[Hb+Mb] amplitude with $\dot{V}$O_*2*peak_ may reflect a higher capacity of hematocrit regulation than for VL. Accordingly, the capacity for diffusive O_2_ conductance may be more important for aerobic exercise performance in some muscles (i.e., RF) compared to others (i.e., VL).

The total[Hb+Mb] concentration at submaximal exercise intensities is greater for HI compared with MID in RF muscle (Figs. 1, 2). It is quite possible that this may also reflect differences in muscle capillarity among individual participants.

### Kinetics of regional muscle deoxygenation pattern

The slope of NIRS variables measured as a function of absolute work (watts) did not differ among participants varying in $\dot{V}$O_*2*peak_ per se (Table 3). Although in the VL muscle each NIRS variable, except S_t_O_2_, plateaued above 70 %W_peak_, in the RF muscle these NIRS variables increased up to $\dot{V}$O_*2*peak_ systematically or at least more rapidly above 70 %W_peak_ than below 50 %W_peak_, except total[Hb+Mb] (Table 3). This suggests that the balance of $\dot{Q}$O_2_/$\dot{V}$O_*2*_ in active muscle is adjusted according to the metabolic requirements of the myocytes as the exercise intensity increases irrespective of the differential $\dot{V}$O_*2*peak_. In previous studies, a controversy has arisen over whether or not the increase in the deoxy[Hb+Mb] slope across normalized power is actually delayed (Boone et al. 2009; Gravelle et al. 2012). In this respect, it is true that, for the whole body, the regression line between $\dot{V}$O_*2*_ and $\dot{Q}$O_2_ is largely independent of training status, age and, perhaps, sex differences (Ogawa et al. 1992; Roca et al. 1992; Proctor et al. 1998). Moreover, in rat muscle the slope of the blood flow to $\dot{V}$O_*2*_ is similar between slow and fast twitch muscle fibers (Ferreira et al. 2006). Thus, it may not be requisite to increase the $\dot{Q}$O_2_/$\dot{V}$O_*2*_ ratio within active muscles to elevate $\dot{V}$O_*2*peak_.

The marked increase in deoxy[Hb+Mb] in RF muscle close to $\dot{V}$O_*2*peak_ suggests that the ability to activate the RF muscle during severe intensity exercise is related to the capacity for high $\dot{V}$O_*2*_′s during cycling exercise (Table 3). Using magnetic resonance T_2_ imaging, Reid et al. (2001) reported that trained subjects had a greater activation of their RF muscle during severe compared with moderate intensity exercise. Moreover, Cannon et al. (2013) showed during incremental knee‐extension exercise that rates of PCr and pH decline varied by muscle region, with one quadriceps muscle often “leading” others. Thus, supplemental activation of other skeletal muscles (e.g., deeper RF and possibly VM, Okushima et al. 2015) may be requisite if the severe intensity exercise is to be continued.

### Methodological consideration

In our preliminary analyses, we fitted NIRS variables with both sigmoidal (Boone et al. 2009; Chin et al. 2011; Spencer et al. 2014) and double linear models (Spencer et al. 2012, 2014; Boone et al. 2016). We found several patterns in the deoxy‐ and total‐[Hb+Mb] responses, for example, a systematic increase, a plateau after reaching the apex, and a decrease after reaching that apex. Therefore we concluded that it was inappropriate to fit a single model to all deoxy‐ and total[Hb+Mb] data. This suggests that other factors besides aerobic capacity also exert an influence over muscle deoxygenation and capillary hematocrit responses, for example, muscle fiber distribution, capillarity, and muscle recruitment patterns. Thus, rather than presuming that one (i.e., sigmoidal) model fits all, future investigations should clarify the disparate patterns of muscle deoxygenation extant during ramp exercise.

### Limitations

The VL and RF muscles measured in this study are two of the dominant locomotor muscles during cycling exercise. These muscles have different EMG activation patterns during incremental cycling (Chin et al. 2011) and their timing of activation is different during a single crank revolution (Heinonen et al. 2015). In addition, there are individual differences in these characteristics due to handle and saddle position, foot position on the pedal, and experience of cycling (Ericson et al. 1985; Takaishi et al. 1998). In our study, we did not measure muscle EMG activity during cycling exercise, and therefore it is not known whether or not the temporal profile of VL and RF activation across the incremental exercise is different among aerobic fitness groups. In addition, we did not assess arterial O_2_ saturation, so we do not know whether exercise‐induced arterial hypoxemia was greater in the HI group compared with MID and LO (Powers et al. 1989; Harms et al. 2000). Should arterial desaturation occur, it could impact the muscle deoxy[Hb+Mb] and S_t_O_2_ responses, potentially contributing to the observed greater muscle deoxygenation in the HI group. The sample size was small for the analysis by two‐way repeated‐measures ANOVA of some (but not all) variables presented in Figures 1, 2. This increases the potential for type II error; therefore the main effect and interaction in each test of Figures 1, 2 may be an underestimate.

## Conclusions

During ramp‐incremental cycling exercise the amplitude of absolute deoxy[Hb+Mb] and S_t_O_2_ for both VL and RF muscles was significantly related to $\dot{V}$O_*2*peak_ (i.e., higher and lower, respectively, in individuals with a greater $\dot{V}$O_*2*peak_). This implies that the capacity for muscle O_2_ extraction, down to the individual active muscles, is correlated with whole‐body aerobic capacity. A high baseline deoxygenation in the RF muscle was negatively associated with $\dot{V}$O_*2*peak_, which implies a greater reliance on increased regional muscle O_2_ delivery during exercise rather than enhanced O_2_ extraction in low‐$\dot{V}$O_*2*peak_ individuals (see Ferreira et al. 2006). In addition, the increase in absolute total[Hb+Mb] (an estimate of diffusive O_2_ conductance) within the RF muscle was significantly correlated with $\dot{V}$O_*2*peak_. These data suggest that the capacity for diffusive O_2_ conductance may be more important for aerobic exercise performance in some muscles (RF) than others (VL). Overall, however, the pattern of muscle $\dot{Q}$O_2_‐to‐$\dot{V}$O_*2*_ balance (i.e., the relative slopes of deoxygenation) and diffusive O_2_ potential (total[Hb+Mb]) were less strongly associated with aerobic capacity, while peak deoxy[Hb+Mb] and S_t_O_2_ (reflecting maximal O_2_ extraction) were more strongly related to $\dot{V}$O_*2*peak_.

## Conflict of Interest

None declared.
